# Progression‐free survival at 2 years post‐autologous transplant: a surrogate end point for overall survival in follicular lymphoma

**DOI:** 10.1002/cam4.1217

**Published:** 2017-10-26

**Authors:** Ana Jiménez‐Ubieto, Carlos Grande, Dolores Caballero, Lucrecia Yáñez, Silvana Novelli, Miguel T. Hernández, María Manzanares, Reyes Arranz, José Javier Ferreiro, Sabela Bobillo, Santiago Mercadal, Andrea Galego, Javier López Jiménez, José María Moraleda, Carlos Vallejo, Carmen Albo, Elena Pérez, Carmen Marrero, Laura Magnano, Luis Palomera, Isidro Jarque, Erika Coria, Antonia Rodriguez, Alejandro Martín, Armando López‐Guillermo, Antonio Salar, Juan José Lahuerta, the GELTAMO (Grupo Español de Linfomas y Trasplantes de Médula Ósea) cooperative study group

**Affiliations:** ^1^ Hospital Universitario 12 de Octubre Madrid Spain; ^2^ Hospital Universitario de Salamanca‐IBSAL Salamanca Spain; ^3^ Hospital Universitario Marqués de Valdecilla Santander Spain; ^4^ Hospital Universitario Sant Pau Barcelona Spain; ^5^ Hospital Universitario de Canarias Tenerife Spain; ^6^ Hospital Universitario de Jerez Jerez Spain; ^7^ Hospital Universitario La Princesa Madrid Spain; ^8^ Hospital Universitario Donostia‐Aránzazu San Sebastián Spain; ^9^ Hospital Universitario Vall de Hebrón Barcelona Spain; ^10^ Hospital Universitario de Bellvitge l'Hospitalet de Llobregat Spain; ^11^ Hospital Universitario A Coruña A Coruña Spain; ^12^ Hospital Universitario Ramón y Cajal Madrid Spain; ^13^ Hospital Universitario Virgen de la Arriaxaca Murcia Spain; ^14^ Hospital Central de Asturias Asturias Spain; ^15^ Hospital Universitario de Vigo Vigo Spain; ^16^ Hospital Universitario Morales de Messeguer Murcia Spain; ^17^ Hospital Universitario Nuestra Señora de La Candelaria Tenerife Spain; ^18^ Hospital Clinic de Barcelona Barcelona Spain; ^19^ Hospital Clínico Universitario Lozano Blesa Zaragoza Spain; ^20^ Hospital Universitario la Fe Valencia Spain; ^21^ Hospital Clínico San Carlos Madrid Spain; ^22^ Hospital del Mar Barcelona Spain

**Keywords:** Autologous Stem Cell Trasplantation, Follicular Lymphoma, Rituximab, PFS 24, CR 30

## Abstract

Overall survival (OS) is the gold‐standard end point for studies evaluating autologous stem cell transplantation (ASCT) in follicular lymphoma (FL), but assessment may be elusive due to the lengthy disease course. We analyzed the validity of two earlier end points, proposed in the setting of first‐line chemo‐/immunotherapy, as surrogates for OS—progression‐free survival (PFS) status at 24 months (PFS24) and complete response at 30 months (CR30) post‐ASCT. We also have investigated the clinical features of patients with early progression after ASCT. Data were available for 626 chemosensitive FL patients who received ASCT between 1989 and 2007. Median follow‐up was 12.2 years from ASCT. In the PFS24 analysis, 153 (24%) patients progressed within 24 months and 447 were alive and progression‐free at 24 months post‐ASCT (26 who died without disease progressions within 24 months were excluded). Early progression was associated with shorter OS (hazard ratio [HR], 6.8; *P *= 0.00001). In the subgroup of patients who received an ASCT in the setting or relapse after being exposed to rituximab, the HR was 11.3 (95% CI, 3.9–30.2; *P *< 0.00001). In the CR30 analysis, 183 of 596 (31%) response‐evaluable patients progressed/died with 30 months post‐ASCT. The absence of CR30 was associated with shorter OS (HR, 7.8; *P* < 0.00001), including in patients with prior rituximab (HR, 8.2). PFS24 and CR30 post‐ASCT are associated with poor outcomes and should be primary end points. Further research is needed to identify this population to be offered alternative treatments.

## Introduction

Follicular lymphoma (FL) is the most common of indolent lymphoma. The combination of conventional chemotherapy, radiotherapy, anti‐CD20 immunotherapy, and stem cell transplantation has contributed to modifying the natural history of FL 1, 2, 3. FL is characterized by a long median overall survival (OS), however, FL remains largely incurable, characterized by repeated remission and recurrence over many years 4. Thus, demonstration of an OS benefit requires a long observation period and large numbers of patients 5, 6. For this reason, the OS assessment to evaluate the efficacy of the different therapeutic alternatives in FL can be elusive. Progression‐free survival (PFS) is presently the principal end point for regulatory approval of new agents. In the pre‐rituximab era, a relationship between higher complete response (CR) rates and improved PFS or OS, was not consistently seen 7, 8. However, in the era of novel targeted therapeutics that offers clinical benefit for long period of times, PFS may as well have fleeting relevance 9, 10. This extended PFS prolongs the duration of clinical trials and limits the ability to approve new effective therapeutic options against FL. Therefore, the identification of alternative or surrogate end points that are measured earlier but can reliably predict PFS treatment effect has been a critical need for decades.

In an attempt to be able to select patients with poor outcome soon in the course of the disease; an observational study of 588 FL patients from the National LymphoCare Study (*NLCS*) treated with the first‐line immunochemotherapy showed that early progression predicted poor outcome 11. After a median follow‐up of 7 years, those with disease progression within 2 years of first‐line treatment had very poor outcomes, with 5‐year OS of 50% compared with 5‐year OS of 90% for patients in the reference group who did not experience an early relapse. Cox regression analysis demonstrated that early relapse was associated with markedly reduced OS with a hazard ratio of 7.17 when compared with the reference group. Additionally, the Follicular Lymphoma Analysis of Surrogacy Hypothesis (FLASH) investigators have recently performed a meta‐analysis of 13 randomized trials of first‐line treatment in FL which included more than 3000 patients treated with a variety of regimens. They found that CR rate at 30 months could predict PFS and proposed this as a possible end point for clinical trials in FL 12.

Autologous stem cell transplantation (ASCT) is commonly used in relapsed follicular lymphoma (FL), having shown an overall survival (OS) benefit in randomized studies 13. However, ASCT is currently rarely used in first remission because randomized studies have shown no OS benefit in this setting, despite some studies demonstrating improved progression‐free survival (PFS) 14, 15, 16, 17, 18. OS is still the gold‐standard end point for studies evaluating ASCT in FL. In the present study, we want to analyze the value of alternative end points associated with prognosis; that have been proposed as surrogates for OS/PFS in the context of first‐line chemo‐/immunotherapy, in a large and lengthy follow‐up series of FL patients from the Spanish GELTAMO database who underwent an ASCT between 1989 and 2007 after chemo or chemoimmunotherapy, either as first or subsequent responses. We also have investigated the clinical features of patients with early progression after ASCT, to better understand its role in FL.

## Patients and Methods

### Study design and participant

The GELTAMO registry database includes 655 patients with nontransformed FL who received ASCT between 1 January 1989, and 31 December 2007 at 44 centers in Spain. All data were updated with a cut‐off date of 31 July 2014. Regarding histology, patients were classified per the Working Formulation into follicular small cleaved cell, follicular mixed small cleaved and large cell, or follicular large cell, and according to the Revised European‐American Classification of Lymphoid Neoplasms as NHL follicular grade 1, 2, or 3 19.

### Response and disease status

Patients’ disease status was classified according to whether they had achieved their first (CR1), second (CR2), or third (CR3) CR, or their first (PR1) or subsequent (PR ≥ 2) partial response (PR) to (immuno)‐chemotherapy at the time of HDT/ASCT. According to GELTAMO guidelines, CR was defined as the disappearance of all clinical evidence of disease, with normalization of X‐rays, CT scans, and laboratory values that had been abnormal before therapy; PR was defined as a ≥50% reduction in measurable disease for ≥1 month; resistant/refractory disease was defined as lymphoma that progressed during initial combination chemotherapy or a response of less than PR to salvage therapy 20, 21. From 1999 onwards, response criteria used were those recommended internationally at the time of HDT/ASCT 22, 23.

### Statistically analysis

Patient‐, disease‐, and transplantation‐related factors were compared between groups using the *Χ* ^2^ test for categorical variables and the Mann‐Whitney test for continuous variables. OS were analyzed using the Kaplan‐Meier method and differences were assessed by the log‐rank test. All *P*‐values were two‐sided and *P *< 0.05 was considered statistically significant. For analysis of PFS24 as a surrogate end point for OS, patients were divided into two groups: those with disease progression within 2 years from ASCT, and those alive without progression at 2 years from ASCT; 26 patients who died without disease progression within 2 years were excluded. In the two groups, OS was measured from time of disease progression and from the 2‐year post‐ASCT landmark, respectively 9 For analysis of CR30, the two groups comprised: those not in CR at 30 months post‐ASCT (due to never achieving or relapsing from CR), and those in CR at post‐ASCT evaluation (~3 months) who had not relapsed at 30 months. In these groups, OS was measured from the time of relapse/progression from CR/PR or death, and from the 30‐month post‐ASCT landmark, respectively. A Cox model was used to calculate the associations between PFS24 status and OS, and CR30 status and OS. To identify factors associated with early POD, logistic regression analysis was performed with backward selection (removing factors when *P* > 0.05).

## Results

Of the 655 patients; there were 203 who underwent ASCT in first complete response (CR), 140 in first partial response (PR), 174 in second CR, 28 in third CR, 81 in second or later PR, and 29 with resistant/refractory disease. These latter patients were excluded, leaving 626 chemosensitive patients for inclusion in these analyses. Demographic and disease characteristics at diagnosis and treatment‐ and transplantation‐related parameters for all 626 patients are summarized in Table 1.

**Table 1:** Main clinical features at diagnosis and treatment variables of the series

Characteristics	Patient series (*n *= 626)
Age at diagnosis, years	
Median (range)	47 (18–73)
≤45	229/622 (37%)
>45	317/649 (63%)
Sex	
Male	307 (49%)
Female	319 (51%)
ECOG performance status^a^	
0–1	485/564 (86%)
≥2	79/564 (14%)
Ann Arbor Stage	
I–II	64/625 (10%)
III–IV	560/625 (90%)
B symptoms	
Absent	435/610 (71%)
Present	175/610 (29%)
Number of nodal sites	
≤4	206/344 (60%)
>4	138/344 (40%)
Bone marrow involvement	
Yes	372/579 (64%)
No	207/579 (36%)
Lactate dehydrogenase^b^	
Normal	395/537 (74%)
High	142/537 (26%)
Tumour mass	
<6 cm	192/449 (43%)
≥6 cm	257/449 (57%)
Hemoglobin level (g/dL)	
≥12	229/299 (77%)
<12	70/299 (23%)
*β*_2_‐microglobulin level^b^	
Low	162/435 (37%)
High	273/435 (63%)
FLIPI score	
Low	104/316 (33%)
Intermediate	115/316 (36%)
High	97/316 (31%)
FLIPI 2 score	
Low	63/301 (21%)
Intermediate	117/301 (39%)
High	121/301 (40%)
Time from diagnosis to HDT/ASCT, years	
≤1	171/609 (28%)
>1	438/609 (72%)
Disease status at HDT/ASCT	
CR1	203 (32.5%)
CR2	174 (28%)
CR3	28 (4.5%)
PR1	140 (22%)
PR ≥ 2	81 (13%)
Received rituximab prior to HDT/ASCT	
Yes	179/592 (30%)
No	413/592 (70%)
TBI‐based conditioning regimen	
Yes	102/625 (16%)
No	523/625 (84%)
Use of PBPC for ASCT	
Yes	492/573 (86%)
No	81/573 (14%)

Data are *n*/*N* (%) unless otherwise stated. In some categories the % values may not sum to 100% due to rounding. BM, bone marrow; FLIPI, Follicular Lymphoma International Prognostic Index; CR, complete response; PR, partial response; ASCT, autologous stem cell transplantation; HDT, high‐dose therapy; TBI, total body irradiation; PBPC, peripheral blood progenitor cells.

a Performance status according to the ECOG scale: 0–1=low level of functional impairment, 2–4 = high level of functional impairment.

b According to normal values of each laboratory.

At data of cut‐off (31 July 2014) median follow‐up for survival from HDT/ASCT was 12.2 years (IQR: 8.1–15.6) and from FL diagnosis was 14.2 years (IQR: 10.9–18). For patients who were rituximab‐naïve prior to HDT/ASCT respective values from the time of ASCT were 14.2 years and 16.4 years, and in patients with prior rituximab respective values were 9.0 and 12.0 years.

Median age at diagnosis was 47 years (range: 18–70), 307 (49%) were male, 97 of 310 (31%) patients with available data had high‐risk FL International Prognostic Index (FLIPI) score, and 121 of 301 (40%) patients had high‐risk FLIPI 2 score. As first‐line therapy 76% of the patients (443/583) received an anthracycline‐based regimen. Prior to HDT/ASCT, 70% of patients received chemotherapy and 30% received chemotherapy plus rituximab. Of 179 patients with previous rituximab exposure (Table 1), 120 received rituximab as first‐line therapy (induction or maintenance) and 101 as part of salvage therapy (induction or maintenance); 42 patients received rituximab in both scenarios. A total of 89 patients underwent ASCT in first response (CR1, *n * =  67; PR1, *n * =  22), and 90 patients underwent ASCT in relapse (CR  ≥ 2, *n* =  67; PR  ≥ 2, *n* =  23) who had been treated with rituximab before transplantation. Median time from diagnosis to HDT/ASCT was 1.9 years (IQR: 0.9–3.5); 28% of patients received HDT/ASCT within 1 year after diagnosis. For transplantation, peripheral blood was used as the progenitor cell source in 86% of patients (median number of CD34+ cells infused was 3 × 10^6^/kg [range: 0.2–37.6]). Only 16% of patients received total body irradiation‐containing conditioning regimens.

A total of 203 (31%) patients underwent ASCT after achieving CR1 (85 of 199 patients in whom data were available [43%] required more than one line of chemotherapy to achieve CR), 174 (27%) were transplanted in CR2, 28 (4%) in CR3, 140 (21%) in PR1 (66 of 134 patients in whom data were available [49%] required more than one line of chemotherapy to achieve PR) and 81 (12%) in PR ≥ 2. Post‐HDT/ASCT disease status was assessable in 640 patients; 585 (91.4%) maintained or upgraded to CR, 32 (5%) maintained or upgraded to PR, and 23 (3.6%) progressed or died. The median duration of first response for patients not transplanted as part of first‐line therapy was 1.4 years (IQR: 0.75–3.1).

In the analysis of PFS24, 153 of 626 (24%) patients progressed within 24 months post‐ASCT, including 62 of 405 (15%) patients who underwent ASCT in CR, and 24 of 179 (13%) patients treated with rituximab‐containing chemotherapy before ASCT. 447 (71%) patients overall, including 325 (80%) who underwent ASCT in CR, and 146 (82%) patients with prior rituximab, were alive and progression‐free at 2 years post‐ASCT, while 26, 18, and 9 patients, respectively, were excluded, having died without disease progression within 2 years. Among patients transplanted at first response (*n *= 343: 203 in CR +140 in PR) o in the setting of relapse (*n *= 283: 174 in CR2 + 28 in CR3 + 81 in PR ≥ 2); a total of 81 of 343 (23.5%) and of 72 of 283 (25.5%) progressed within 24 months post‐ASCT (*P* > 0.5). 248 and 199 were alive and progression‐free at 2 years post‐ASCT, while 14 and 12 patients were excluded, having died without disease progression within 2 years; respectively. In the subgroup of patients transplanted and relapsed after a rituximab regimen only 11 of 87 (*n *= 13%) progressed within the first 2 years post‐ASCT.

For the 153 patients who progressed within 24 months post‐ASCT, the median age at diagnosis was 48 years (range, 28–70 years), and 60% of patients were male. Patients who progressed within 24 months post‐ASCT were more likely to be male (93/153 vs. 207/447; *P*=.002), to have at diagnosis an Eastern Cooperative Oncology Group (ECOG) Performance Status (PS) >1 (28/141 vs. 47/298; *P *= 0.007), bone marrow infiltration (96/144 vs. 201/413; *P* = 0.0005), high lactate dehydrogenase (LDH) level (45/136 vs. 91/38; *P* = 0.04) and more than four nodal areas affected (40/80 vs. 91/246 *P *= 0.04). There were also fewer patients who received rituximab before ASCT in the subgroup of patients who progressed within 24 months post‐ASCT (24/143 vs. 146/423; *P* < 0.00001). For patients undergoing ASCT in 2nd/3rd CR or PR ≥ 2; there were more patients with a duration of first remission less than 12 months in the subgroup of patients who progressed within 24 months post‐ASCT (27/47 vs. 42/136; *P *= 0.001). In the multivariate analysis, factors associated with early POD were poor performance status (≥2) (*P *= 0.0005) and the absence of rituximab before ASCT (*P *= 0.002).

**Figure 1:**
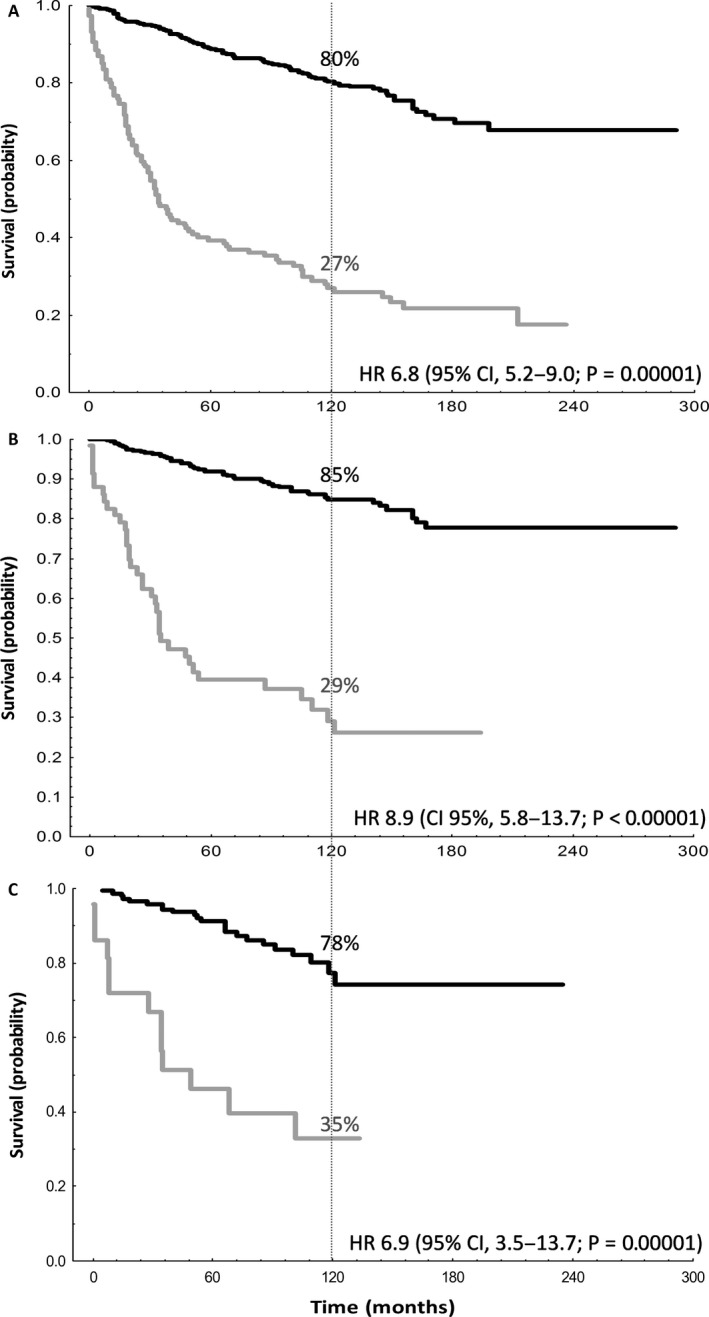
Evaluation of OS according to PFS24 status post‐ASCT. Disease progression within 24 months from ASCT was associated with poorer OS, in (A) the overall population, (B) patients who underwent ASCT in CR, and (C) patients who received rituximab prior to ASCT. Black line: Patients alive and progression‐free at 24 months post‐ASCT; OS measured from landmark of 24 months. (A) *n *= 447, (B) *n *= 325, (C) *n *= 146. Gray line: Patients who progressed within 24 months post‐ASCT; OS measured from time of progression. (A) *n *= 153, (B) *n *= 62, (C) *n *= 24.

Early progression was associated with shorter OS. In all the patients (*n *= 626), OS from a risk‐defining event at 5 years in the patients who progressed within the 24 months post‐ASCT was 39%, and at 10 years was 27% compared with 89% and 80%, respectively, for the reference group. The hazard ratio (HR) for death was 6.8 (95% confidence interval [CI], 5.2–9.0; *P *= 0.00001) (Fig. 1A); among patients transplanted in CR (*n *= 405), the HR was 8.9 (95% CI, 5.8–13.7; *P *< 0.00001) (Fig. 1B), and for patients transplanted in PR (*n *= 221) the HR was 3.9 (95% CI, 2.6–5.8; *P *< 0.00001). In patients transplanted in the setting of relapse (*n *= 283); OS from a risk‐defining event at 5 years in the patients who progressed within the 24 months post‐ASCT was 31%, and at 10 years was 22% compared with 85% and 75%, respectively, for the reference group; with an HR of 6.5 (95% confidence interval [CI], 3.7–8.71; *P *= 0.00001). Finally, in patients with prior rituximab (*n *= 179) the HR was 6.9 (95% CI, 3.5–13.7; *P *= 0.00001) (Fig. 1C); this association of PFS24 with OS was seen independent of patients being transplanted in first response (HR, 5.0 [95% CI, 1.8–13.5; *P *< 0.00001), or in the setting of salvage therapy (*n *= 87) (HR, 11.3 [95% CI, 3.9–30.2; *P *< 0.00001).

To further characterize the effect of progression within the first 2 years after ASCT, OS from time of progression was evaluated for patients whose disease progressed early versus patients whose disease progressed more than 2 years after ASCT. Patients with early progression had inferior OS compared with those progressions occurred after 2 years (HR, 1.9; 95% CI, 1.45 to 2.5; *P = *0.000006).

We have further information on 220 patients who relapsed post‐ASCT. A total of 24 patients were consolidated with an allogenic stem cell transplantation. Of them, 12 patients (50%) are still alive and free of FL at last follow‐up. The causes of death of the remaining 12 patients were progression of FL in four cases, infection in two cases, graft versus host disease in four cases, and non‐specified non‐relapsed mortality in two cases.

**Figure 2:**
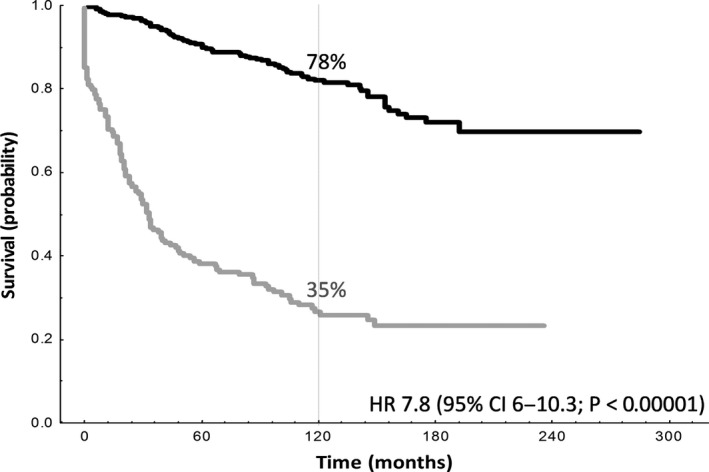
Evaluation of OS according to CR30 status post‐ASCT. Patients who were not in CR at 30 months post‐ASCT had poorer OS. Black line: Patients in CR at 30 months post‐ASCT; OS measured from landmark of 30 months (*n *= 413). Gray line: Patients not in CR at 30 months post‐ASCT; OS measured from time of relapse/progression or death (*n *= 183).

For the analysis of CR30, 616 patients had response assessments post‐ASCT: 567 (92%) were in CR, 29 (5%) were in PR and 20 (3%) progressed or died. In 563 patients with available data, 175 (31%) received rituximab prior to ASCT, of whom 134 were transplanted in CR and 89 while in first response. Of 596 patients with CR/PR post‐ASCT, 183 (31%) progressed or died within 30 months post‐ASCT. The absence of CR in 30 months was associated with shorter OS: the HR for death was 7.8 (95% CI, 6.0–10.3; *P* < 0.00001) (Fig. 2). This association was retained in patients who received rituximab before ASCT (HR, 8.2 [95% CI, 4.5–15.1; *P* < 0.00001).

## Discussion

In aggressive lymphomas, such as diffuse large B‐cell lymphoma, early relapse post‐ASCT is widely accepted as associated with poor prognosis 24. However, in FL there is only one study that demonstrates that an early relapse post‐ASCT has a negative impact on OS 25. This report represents the first analysis of chemosensitive FL patients receiving ASCT that suggests that PFS24 could be a valid surrogate and primary end points for OS in this setting. The findings from our large patient series offer validation of previously published data outside ASCT. Casulo et al. 11 have identified a subgroup of FL patients treated with chemoimmunotherapy who progressed within the first 2 years after diagnosis and who are at high risk for death after treatment. Another study 26 has recently confirmed this data in a 7.7 years median‐follow cohort of 94 patients with FL who also received first‐line chemoimmunotherapy .

Additionally, it has been demonstrated in a large series of 920 newly diagnosed FL that event‐free survival (EFS) at 12 and 24 months from diagnosis in patients treated with first‐line chemoimmunotherapy is an extremely powerful prognostic indicator of outcome in FL 27. Our results show that persistence of complete response at 30 months after ASCT could as well be a surrogate for OS in FL who are autografted. These results are consistent with the data obtained by Shi Q et al. 12 in a pooled analysis of randomized chemotherapy, immunotherapy, or chemoimmunotherapy trials that demonstrated that CR at 30 months after initiation of induction treatment may serve a surrogate end point for PFS in the setting of first‐line FL treatment trial.

From this study, some clinical characteristics at diagnosis such as older age, male sex, ECOG PS > 1, bone marrow infiltration, high lactate dehydrogenase level (LDH) or more than four nodal areas affected were associated with progression within 24 months post‐ASCT and, therefore, with poor outcome. Duration of first remission less than 12 months also predicted for an early relapse. Murakami et al. 26 also identified high LDH levels as a predictor of early death in patients treated with first‐line chemoimmunotherapy. Furthermore, only ECOG PS>1 and the fact of being rituximab‐naïve at the moment of ASCT had statistical significance in the multivariate analysis. Neither the moment (1st or subsequent responses) nor the status of the disease at ASCT (CR or PR) was associated with the incidence of early progression. The fact that an early relapse after ASCT in rituximab exposed patients seems to be less frequent than in rituximab‐naive, has already been demonstrated in diffuse large B‐cell lymphoma 24. Nevertheless, in our series, there are still 13% of patients who will have an early relapse post‐ASCT after a rituximab‐based therapy; even when transplanted in CR1. A positive impact of rituximab sensitivity on the results of ASCT has been recently suggested by the Hutchinson Cancer Research Center in 35 relapsed FL patients 28, however, the poor outcomes of patients who relapse soon after ASCT in the rituximab era has not been described.

We agree with Casulo et al. 11 with the idea that ASCT may potentially abrogate the negative prognostic effect of early relapse after a chemoimmunotherapy regimen because the salvage chemotherapy followed by ASCT has an established role in relapsed FL 13, 29, 30, 31. Nevertheless, from our data, we can conclude that although ASCT at first relapse could be a reasonable option in some high‐risk FL patients; there are still FL populations in whom consolidation with allogenic stem cell transplantation or new therapeutic options are needed. Additionally, there are some patients autografted in CR1 after chemo or chemoimmunochemotherapy, who will die soon after ASCT. The identification of these patients soon during the course of the disease would be of capital interest. Some genetic alterations, such as chromosomal gains and losses or rearrangements of P53 or MYC, have been implicated in poor outcomes in FL 32, 33. Additionally, novel biomarkers that incorporate both clinical and genetic determinants of poor risk are being developed with the hope of identifying high‐risk patients at diagnosis to offer biologically rational targeted therapies. Recently it has been published in *Lancet Oncology* that the integration of the mutational status of seven genes (EZH2, ARID1A, MEF2B, EP300, FOXO1, CREBBP, and CARD11) by DNA deep sequencing with clinical risk factors improves prognostication for patients with follicular lymphoma receiving first‐line immunochemotherapy 34.

The clinical implications of early progression after ASCT are highly relevant to improve patient survival. This issue is of especial interest in patients who are autografted at relapse after a chemoimmunotherapy rescue regimen, because this strategy remains a standard of care nowadays. In this population, a progression during the first 24 months from ASCT carried an increased risk of death with a hazard ratio of 11.3 and the absence of CR at 30 months post‐ASCT was associated with shorter OS with and HR of 8.1. The effect of early progression after ASCT with novel‐chemotherapy‐containing regimens will require validation in future studies.

Based on our data, we cannot recommend PFS 24 or CR 30 as a better end point in FL patients receiving an ASCT. As PFS 24 is an end point that can be evaluated in a shorter period of time since ASCT, and probably with less exigent criteria; we feel that PFS24 could be used as the preferred end point. Nevertheless, it is not clear, and efforts should persist in the rational design of clinical trials that risk stratify FL patients undergoing ASCT and evaluate novel end points in this disease.

Some limitations of our analysis should be noted, specifically the retrospective design and the fact that histologic results were not centrally reviewed. However, we believe these issues are not likely to have affected our conclusions because previous data have suggested a high degree of accuracy in community diagnosis of FL.

Based on our findings, we propose that PFS24 and CR30 should be used as primary end points and as surrogates for OS in studies of FL patients receiving ASCT. Disease progression within 2 years and absence of CR at 30 months post‐ASCT define new high‐risk groups who should be ideally identified soon in order to be offered different therapeutic strategies, such as allogenic stem cell transplantation or novel drugs. Different diagnostic tools such as means of targeted sequencing or gene expression profiling would be essential to select this high‐risk group of patients and to improve their outcome.

## Conflict of Interest

None declared.
